# Retrograde intrabronchial suturing of a latissimus dorsi flap for the surgical repair of bronchopleural fistula: a case report

**DOI:** 10.1093/jscr/rjaf1057

**Published:** 2026-01-08

**Authors:** Akifumi Nakamura, Nobuyuki Kondo, Masaki Hashimoto, Soichiro Funaki

**Affiliations:** Division of Thoracic Surgery, Department of Surgery, Hyogo Medical University, 1-1 Mukogawa-cho, Nishinomiya, Hyogo 663-8501, Japan; Division of Thoracic Surgery, Department of Surgery, Hyogo Medical University, 1-1 Mukogawa-cho, Nishinomiya, Hyogo 663-8501, Japan; Division of Thoracic Surgery, Department of Surgery, Hyogo Medical University, 1-1 Mukogawa-cho, Nishinomiya, Hyogo 663-8501, Japan; Division of Thoracic Surgery, Department of Surgery, Hyogo Medical University, 1-1 Mukogawa-cho, Nishinomiya, Hyogo 663-8501, Japan

**Keywords:** bronchopleural fistula, latissimus dorsi flap, intrabronchial

## Abstract

Bronchopleural fistula (BPF) is a rare but serious postoperative complication after lung surgery. While secondary procedures are often required, primary closure with autologous tissue may be feasible in selected cases. A 71-year-old man developed a large BPF after left upper lobectomy, associated with steroid-treated acute exacerbation of interstitial pneumonia. Computed tomography (CT) and bronchoscopy revealed complete bronchial stump dehiscence with a 10-mm defect. As no infection was present, elective surgery was performed 6 months later. Using bronchoscopic guidance, a pedicled latissimus dorsi muscle flap was retrogradely inserted and sutured into the bronchus. The postoperative course was uncomplicated, and CT and bronchoscopy at 3 and 6 months confirmed complete closure and healing. Retrograde bronchial filling using a latissimus dorsi flap with bronchoscopic assistance achieved successful closure of a large, non-infected chronic BPF and may be a useful option in selected patients.

## Introduction

Bronchopleural fistula (BPF) is a serious postoperative complication following lung surgery [1]. Management strategies for BPF depend on the patient’s overall condition, the presence of infection, the size and location of the fistula, and the timing of its onset [2, 3]. We present a case of a large, non-infected BPF successfully treated by retrograde bronchial filling using a pedicled latissimus dorsi flap.

## Case report

A 71-year-old man undergoing steroid therapy for rheumatoid arthritis was admitted to our hospital. A follow-up chest radiograph revealed an abnormal shadow, and computed tomography (CT) subsequently identified a solid tumor (maximum diameter, 1.9 cm) in the left upper lobe. A transbronchial biopsy confirmed squamous cell carcinoma, leading to a clinical diagnosis of lung squamous cell carcinoma (c-T1bN0M0, stage IA2). The patient underwent video-assisted thoracoscopic left upper lobectomy. Owing to a severely incomplete fissure, a bronchial-first approach was adopted. The operation lasted 103 min with an estimated blood loss of 10 mL. Postoperatively, the patient experienced an acute exacerbation of interstitial pneumonia, which was managed with a steroid pulse followed by prednisolone. He was discharged 1 month after surgery on home oxygen therapy, with his prednisolone dose gradually tapered during follow-up. Final pathology revealed adenosquamous carcinoma (p-T1bN0M0).

**Figure 1:**
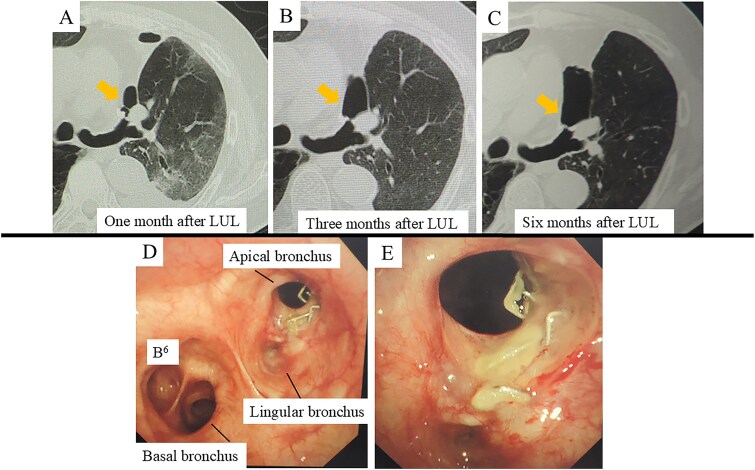
CT and bronchoscopic findings post-left upper lobectomy (LUL). (A–C) CT scans obtained at 1, 3, and 6 months postoperatively. Panel A shows a small airspace (gold arrow) near the upper segment of the bronchial stump that progressively enlarges over time. (D–E) Bronchoscopic images at 6 months demonstrate failure of the staple line for the upper division bronchus (B1 + 2, B3) at the upper lobe bronchial stump, resulting in an ~10 mm complete separation with surrounding whitish changes.

At 1 month postoperatively, CT imaging revealed a small air space adjacent to the upper lobe bronchial stump (Fig. 1A). In the absence of infection, conservative observation was chosen. Despite the patient remaining asymptomatic, subsequent CT scans at 3 and 6 months demonstrated gradual enlargement of the air space (Fig. 1B and C). Bronchoscopic evaluation showed failure of the staple line at the upper division bronchus (B1 + 2, B3) near the bronchial stump, resulting in a complete separation of ~10 mm with surrounding whitish changes (Fig. 1D and E). An infection-free upper lobe BPF was diagnosed, and a one-stage closure using a latissimus dorsi flap was planned.

**Figure 2:**
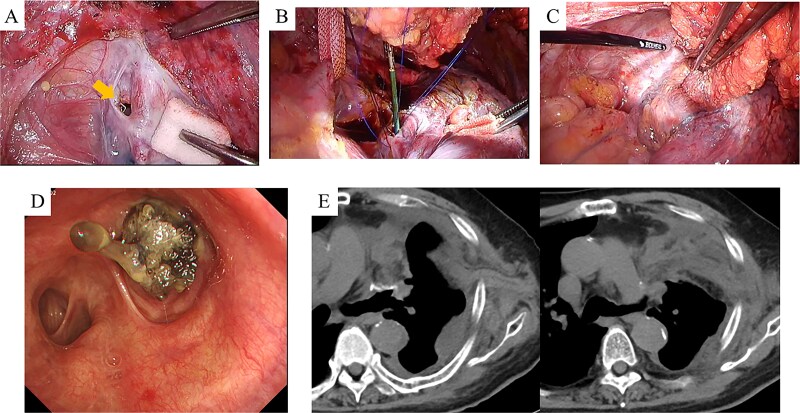
Intraoperative and early postoperative findings. (A–C) Panel A identifies the fistula at the upper lobe bronchial stump (arrow), adjacent to the pulmonary artery. Panel B shows a bronchoscope being used to guide the biopsy forceps from the fistula site into the thoracic cavity to grasp the latissimus dorsi muscle flap. Panel C shows the flap secured into the bronchial lumen in a retrograde fashion, with fibrin glue applied for reinforcement. (D) Bronchoscopic view on postoperative day 7 confirms visibility of the latissimus dorsi muscle flap within the bronchial lumen. (E) CT scans on postoperative day 7 demonstrate secure suturing of the latissimus dorsi muscle flap to the fistula site and resolution of the preoperative airspace.

**Figure 3:**
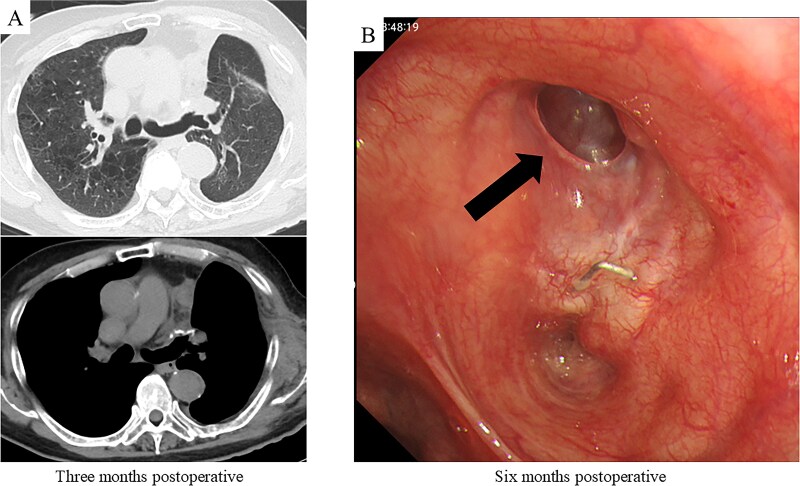
Postoperative CT imaging and bronchoscopic evaluation. (A) CT scans at 3 months postoperatively reveal no recurrence of air leakage, indicating a stable clinical course. (B) Bronchoscopic examination at 6 months postoperatively shows satisfactory granulation tissue formation at the upper lobe bronchial stump, consistent with complete healing.

After harvesting the latissimus dorsi flap through a posterolateral incision, a thoracotomy was performed. Intrathoracic adhesions were dissected to expose the bronchial stump fistula, which measured ~10 mm (Fig. 2A); no evidence of empyema was observed. Given the large, deep configuration of the fistula, which would complicate delivery and complete coverage of the flap, a bronchoscope was inserted and biopsy forceps were advanced through the fistula into the thoracic cavity. The latissimus dorsi flap was grasped with the forceps, guided into the thoracic cavity, and secured in place with sutures (Fig. 2B). Fibrin glue was applied around the repair site for reinforcement (Fig. 2C). After confirming the absence of any air leak, the procedure was completed (Supplementary video 1). The operation lasted 140 min, with an estimated blood loss of 20 ml. The postoperative course was uneventful. On postoperative day 7, CT and bronchoscopy confirmed that the latissimus dorsi muscle had retrogradely filled the bronchial lumen and that the fistula was completely closed (Fig. 2D and E). The patient was discharged 13 days postoperatively, and home oxygen therapy was discontinued. Follow-up CT scans at 3 months showed gradual retraction of the latissimus dorsi muscle from the bronchial lumen, yet no residual air space was observed (Fig. 3A). At 6 months, bronchoscopy revealed satisfactory granulation tissue formation at the upper lobe bronchial stump, indicating complete healing (Fig. 3B).

## Discussion

Despite significant advancements in surgical techniques and perioperative management, BPF continues to pose a formidable challenge for thoracic surgeons. Treatment of BPF requires thorough debridement of the pleural cavity followed by definitive closure of the fistula. For small fistulas (5 mm or less), where adequate drainage is achievable and the patient is in good condition overall, bronchoscopic closure may be attempted. In contrast, fistulas >6 mm or those associated with a necrotic bronchial stump generally warrant surgical repair [2]. In such cases, surgical closure is best delayed until the thoracic cavity is completely cleansed and the patient’s clinical status improves, often necessitating a secondary procedure such as thoracostomy or thoracoplasty. Alternatively, if infection is well controlled and the patient remains stable, primary surgical closure may be considered. Following thorough debridement, the bronchial stump is typically sutured and closed, with established techniques involving coverage of the suture line and obliteration of the empyema cavity using a pedicled omental or latissimus dorsi flap [1, 2].

In some instances, primary suture closure is not feasible. In these situations, packing the fistula with omental tissue and covering the surrounding area has been reported [4]. Sutured closures may be exposed to mechanical tension, potentially impairing blood flow, and narrowing of the bronchial lumen during closure can predispose to atelectasis and pneumonia. Utilizing omental tissue in direct contact with the bronchial mucosa can mitigate these concerns by preserving blood flow and leveraging the omentum’s anti-inflammatory properties to promote mucosal regeneration [3]. A critical aspect of this method is ensuring broad contact between the autologous tissue and bronchial mucosa [4]. The filling tissue must be secured in a manner that preserves bronchial blood flow while being circumferentially anchored to adjacent structures to prevent recurrent air leaks, often with the reinforcement of fibrin glue. Care must be taken to avoid occluding the residual bronchus. Intraoperative bronchoscopy is then employed to confirm that the bronchus remains patent and free of air leaks under pressure.

In this case, it was challenging to directly suture the large fistula involving the entire upper division bronchus because of its size and adhesion to the pulmonary artery. The absence of infection and chronic course allowed for a primary closure approach. Instead of omental tissue, a latissimus dorsi muscle flap was used to minimize operative time, considering the patient's history of interstitial pneumonia. The muscle flap was advanced into the bronchial lumen using forceps under bronchoscopic guidance. Securing the flap with sutures provided stable fixation, promoted epithelialization, and allowed complete healing. This technique may represent a feasible treatment option for similar cases.

## Supplementary Material

supplemental_video_rjaf1057
